# A case report of implanted bone displacement after abscess formation

**DOI:** 10.1097/MD.0000000000042241

**Published:** 2025-04-25

**Authors:** Xiao Qi Chen, Shu Ping Ma, Yue Han Li, Shi Hang Xu

**Affiliations:** a Xiang’an Hospital of Xiamen University, Xiamen, China; b Children’s Hospital of Chongqing Medical University, Chongqing, China

**Keywords:** chronic osteomyelitis, implanted bone, implant-related infection, MRSA

## Abstract

**Rationale:**

Implanted bone is a commonly used filling material for bone defects, participating in the formation of new bone. Complications associated with implanted bone include nonunion, infection, etc. Few cases of implanted bone displacement through gravitational abscesses have been reported in the literature. This article aims to improve clinicians’ recognition of this phenomenon and discuss preventive measures and treatment strategies for implant-associated infections.

**Patient concerns:**

A 7-year-old female patient was admitted to the hospital due to recurrent purulent fluid discharge from the incision site for 3 months following a right hip surgery 4 months ago. Wound secretion culture revealed a methicillin-resistant *Staphylococcus aureus* infection. The imaging before the first debridement in the external hospital showed that there were 2 high-density shadows around the right femoral greater trochanter, which were implanted bone.

**Diagnoses:**

Implant-related infection and chronic osteomyelitis.

**Interventions:**

The implant and inflammatory lesions were removed through multiple surgeries and anti-infection treatment.

**Outcomes:**

After 6 months of follow-up, there was no sign of infection in the wound.

**Lessons:**

It is rare-reported that implanted bone displacement is caused by factors such as abscess formation. Treatment and understanding of implanted bone around the greater trochanter of the right femur is important. Early recognition and surgical removal of displaced implanted bone play a crucial role in infection control and disease prognosis.

## 1. Introduction

Implanted bone is wide-used in the treatment of trauma, degenerative diseases, infections, and tumors. Annually, at least 2.2 million surgeries require implanted bone, with an infection rate of 2.03%.^[1,2]^ However, it is rare-reported that implanted bone displacement caused by factors such as abscess formation. Early recognition and surgical removal of displaced implanted bone play a crucial role in infection control and disease prognosis. On October 8, 2023, Xiang’an Hospital of Xiamen University admitted a child with an implant-related infection following surgery for developmental dysplasia of the hip. The child’s implanted bone had shifted along the infected cavity which is reported as follows.

## 2. Case presentation

**Figure 1.**
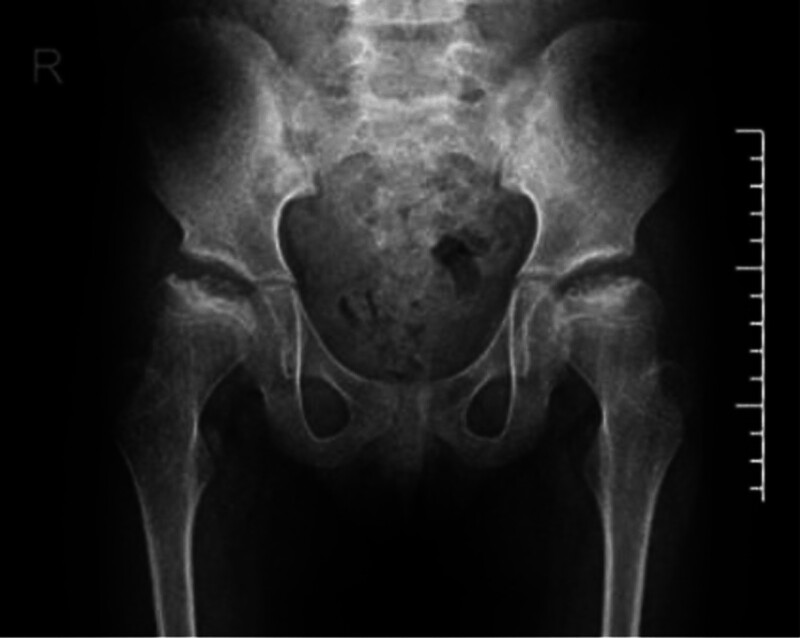
The initial X-ray findings prior to the first operation were consistent with bilateral femoral head necrosis.

**Figure 2.**
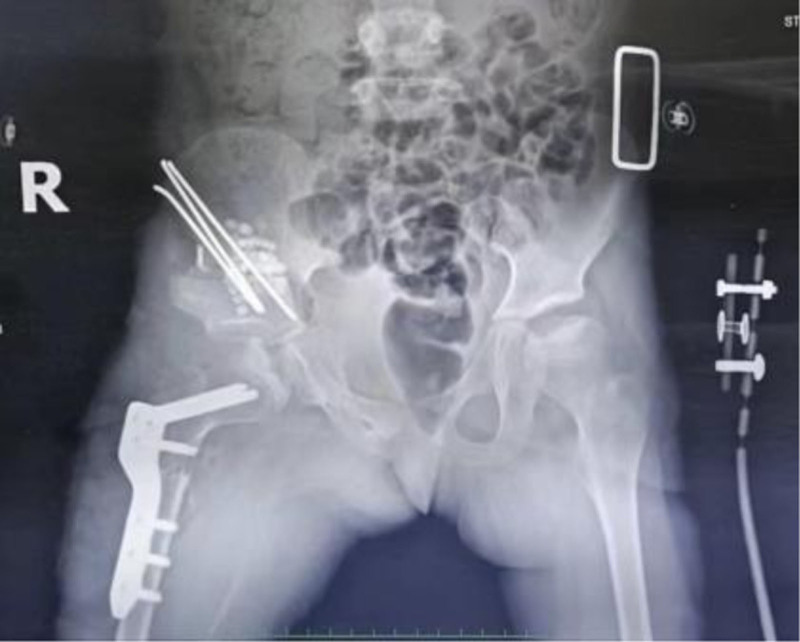
The initial X-ray findings after the first operation were consistent with femoral plate fixation, right iliac K-wire fixation, and the placement of implanted bone.

**Figure 3.**
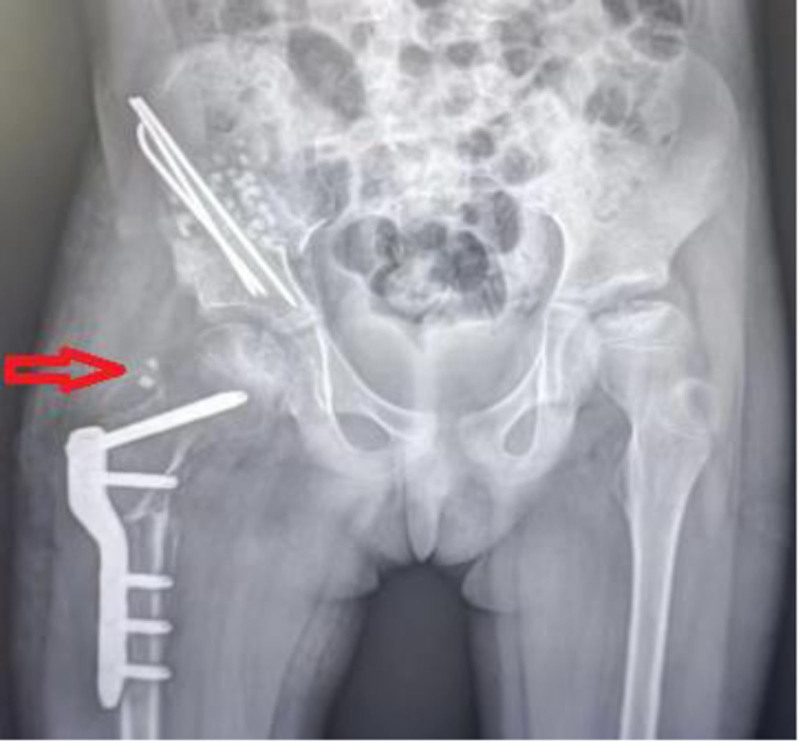
The initial X-ray findings after the first debridement showed 2 high-density shadows around the right femoral greater trochanter, which correspond to the implanted bone (indicated by the red arrow).

A 7-year-old female patient was admitted to the hospital due to recurrent purulent fluid discharge from the incision site for 3 months following a right hip surgery 4 months ago. The patient reported no significant medical history, family history, psychosocial issues, comorbidities, or relevant genetic information. But she was allergic to various antibiotics, seafood, and mangoes. The patient underwent a right femoral rotational osteotomy, right iliac osteotomy, acetabular plasty, and iliac bone grafting at hospitals in Shanghai on June 19, 2023, due to right developmental dysplasia of the hip. During the surgery, implanted bone was implanted at the iliac osteotomy site and fixed with a Kirschner wire, while the femoral rotational osteotomy was stabilized with a steel plate. Figures 1 and 2 show the preoperative and postoperative radiological findings of this surgery. Three weeks after the surgery (2 weeks after discharge, July 10, 2023), recurrent purulent fluid was observed from the surgical incisions on the right iliac region and right thigh, with the purulent fluid from the iliac incision preceding that of the thigh incision. Two debridements were performed at an external hospital for the infection sites, but no improvement was noted. Wound secretion culture revealed a methicillin-resistant Staphylococcus aureus (MRSA) infection. Figure 3 depicts the radiological findings before the first debridement at the external hospital, revealing 2 high-density shadows (implanted bone) around the right femoral greater trochanter.

**Figure 4.**
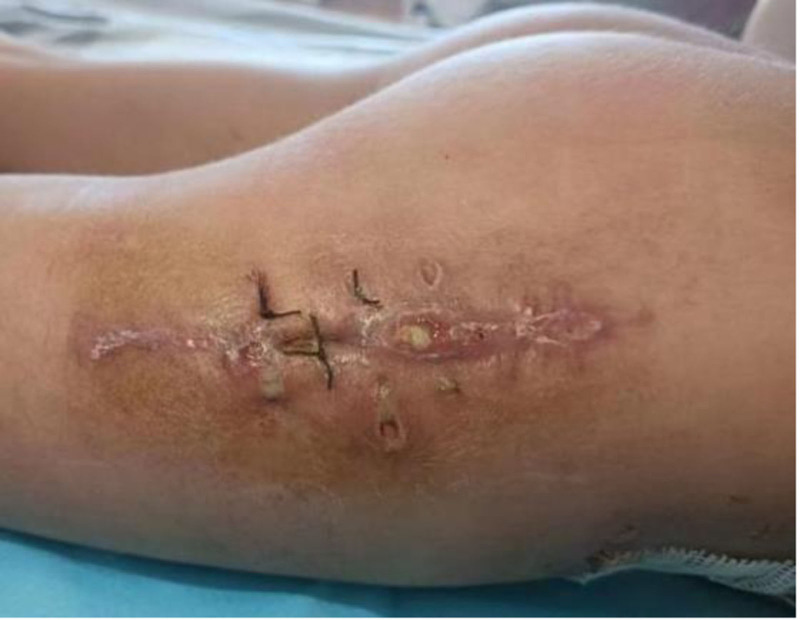
Before the first surgery at our hospital, the right femoral incision had been broken and was discharging pus.

**Figure 5.**
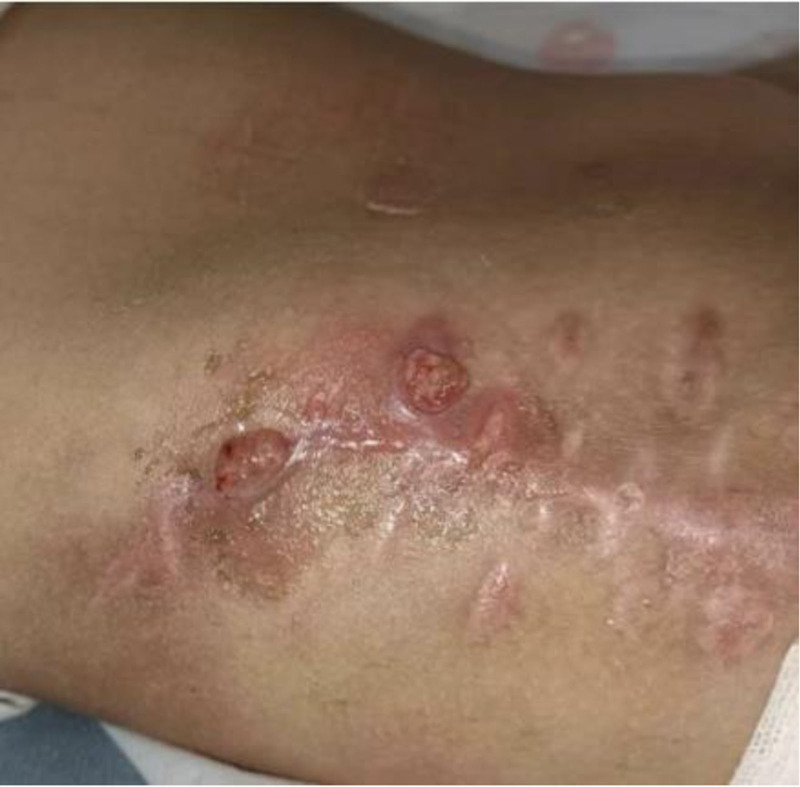
Before the first surgery at our hospital, the right iliac incision was already broken and discharging pus.

After visiting several hospitals, the patient was admitted to our hospital on October 8, 2023. Physical examination revealed and a sinus tract, with a diameter of approximately 0.8 cm, discharging similar pus at the lateral right thigh incision (Fig. 4). Two additional sinus tracts, each with a diameter of approximately 1.0 cm, discharging yellowish-white pus at the right iliac surgical (Fig. 5). Radiological imaging indicated that callus formation was satisfactory in the right iliac bone but not in the right femoral bone. Considering that the high-density shadows around the greater trochanter of the femur were implanted bone, and that the implanted bone was in a locally encapsulated state, this surgery would not handle it temporarily. Given that the recurrent pus discharge was attributed to the infected iliac implant, we decided to perform an incision and drainage of abscesses in the lateral right thigh and right iliac regions, removal of the internal fixation and implanted bone from the right iliac area, excision of inflammatory lesions, rinsing and drainage of the wound cavity on October 19, 2023, at our hospital. During the operation, a bone bridge was observed around the lateral iliac osteotomy site, with local bone destruction forming a cavity extending into the iliac fossa. The implanted bone was distributed on both sides of the iliac bone, which was surrounded by chronic granulation tissue. Postoperative diagnosis: chronic osteomyelitis of left ilium, implant-related infection. After the operation, the wound cavity was rinsed and drained for 2 weeks. The patient received a 3-week course of vancomycin therapy for anti-infection treatment. Three weeks after the surgery, an ultrasound of the operation area indicated no significant fluid collection, and the drainage tube was subsequently removed before the patient’s discharge.

On November 28, 2023, 18 days after the last discharge, the patient’s family noticed pus flowing under the right iliac surgical incision once again. An ultrasound examination indicated localized fluid collection below the right iliac surgical incision, a bone fragment at the distal end of the sinus tract, and a pinprick-sized wound on the right thigh incision containing a small amount of fluid extending to the steel plate. No significant fluid collection was noted in the iliac operation area. The patient was readmitted for treatment. The wound secretion culture also confirmed MRSA infection. After a multidisciplinary discussion, the following conclusions were drawn: the patient’s current diagnosis was implanted bone infection and chronic osteomyelitis. As indicated by imaging, the patient exhibited good callus formation in the right femoral bone, and it was deemed appropriate to remove the steel plate, K-wire, and implanted bone. The wound was left open for dressing changes to facilitate the healing process. An external fixation system was applied to the affected limb to provide stability. Teicoplanin and ceftazidime were administered for anti-infection treatment. After the wound infection was controlled, the quadriceps femoris or tensor fasciae latae muscle flap was planned to be used to cover the wound to prevent infection and promote healing. The patient underwent surgical treatment on December 8, 2023, at our hospital. Intraoperatively, we observed a fibrous membrane encapsulating the right femoral steel plate, with a significant amount of inflammatory granulation tissue within the membrane. Some steel nails were loose, and 2 residual implanted bone blocks were found approximately 1.0 cm beneath the skin at the sinus tract of the right iliac incision. The steel plate, surrounding fibrous membrane, and granulation tissue were completely removed, and drainage tubes were placed. The surrounding inflammatory granuloma, sinus tract, and implanted bone blocks were excised along the sinus tract, and the drainage tube was retained in the wound. Given the complete excision of the fibrous membrane, sinus tract, and granulation tissue, we decided to perform a one-stage suture. The patient was discharged after 3 weeks of anti-infection treatment with teicoplanin following the surgery. During the 2-month follow-up, no sign of wound rupture or pus discharge was observed.

**Figure 6.**
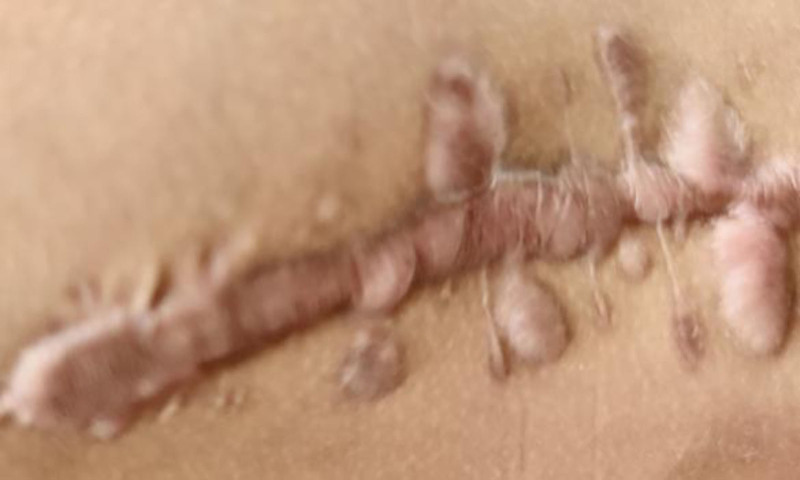
The incision on the right thigh showed no local inflammatory manifestations 10 months after the last surgery at our hospital.

**Figure 7.**
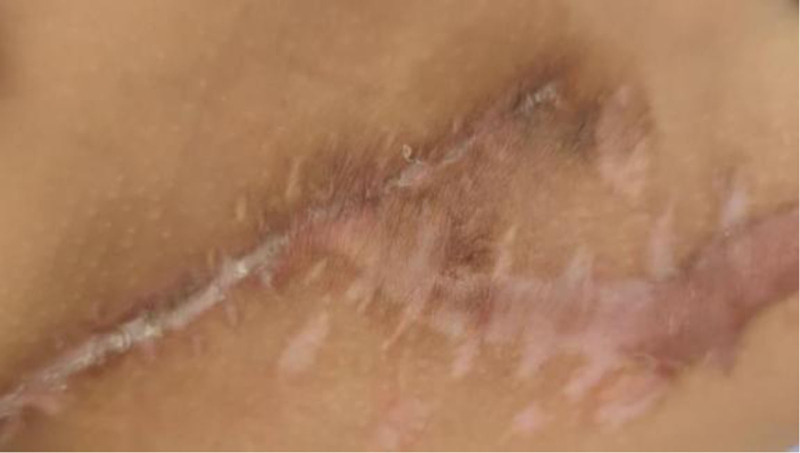
The right iliac incision, 10 months after undergoing the last surgery at our hospital, exhibited no signs of local inflammation.

On February 25, 2024, the patient’s right iliac incision ruptured again, discharging pus and dead bone fragments. An ultrasound examination indicated inflammatory changes in the soft tissue at the right iliac-lumbar region, with punctate and striated echoes of fragmented bone fragments inside. This was considered to be caused by chronic osteomyelitis of the right iliac bone. Therefore, the patient underwent debridement of the right iliac wound on March 7, 2024. Intraoperatively, dead bone fragments were found on the medial side of the right iliac bone. We removed the dead bone fragments along with the surrounding inflammatory tissue. Since the patient experienced an allergic reaction after using vancomycin this time, we switched to linezolid for anti-infective treatment.The patient was discharged after a 3-week course of intravenous linezolid anti-infection treatment following the surgery. The patient continued to take linezolid orally for 2 months after discharge. During the multiple surgeries and other treatments conducted in our hospital, the patient and her family actively cooperated with and trusted us. After nearly 10 months of follow-up (November 14, 2024), there was no sign of infection in the wound, as seen in Figures 6 and 7. MRI showed that the signal intensity of the right iliac bone marrow edema was significantly reduced in comparison with the previous one, and the inflammatory changes around the right iliac bone and right femur were reduced in size compared to before.

## 3. Discussion

Implanted bone demonstrates exceptional biocompatibility, aiding in bone healing through processes such as osteogenesis, osteoconduction, and osteoinduction. Consequently, it serves as an effective means of replacing or repairing damaged, missing, or diseased bone tissues throughout the body. Its applications are wide, encompassing fracture repair, bone defect restoration, and the treatment of bone joints and tumors. Literature on implanted bone reports a range of complications, including early resorption during the healing process, infection, nonunion, rejection, thrombosis, nerve damage, and aseptic loosening, among others.^[3,4]^ Purulent secretions, wound dehiscence, and wound ulceration are all unmistakable signs of infection.^[5]^ Graft-related infections have been thoroughly documented.^[6,7]^ However, cases involving migrated infected implants are infrequently reported, posing a challenge for early identification. In this particular case, we noticed the displacement of the implanted bone by comparing imaging data from before and after surgery. As the disease progressed, the infected implant developed a sinus tract, exhibiting symptoms of infection. With the aid of color Doppler ultrasound, the diagnosis of an infected and migrated implant was unequivocally established.

Considering the patient’s subsequent clinical changes and treatment outcomes, the implanted bone should have been removed during the initial surgery at our hospital. The displacement of the implanted bone to the lateral side of the right femoral greater trochanter was one of the causes of the disease’s persistence. If the child’s imaging data is not thoroughly compared, there is a high likelihood that the implanted bone, which has been displaced to the outer side of the right femoral greater trochanter, will be overlooked. This case has 2 special features. Firstly, the implanted bone moved along the abscess for a long distance. After loosening due to post-infection, the implanted bone shifted towards the greater trochanter of the right femur but was halted by the tensor fasciae latae and quadriceps femoris muscles. The implanted bone was unable to drain along with the purulent fluid, which hindered the healing process. Secondly, the relationship between the migrated implant and wound infection is intriguing. The alternating infection manifestations between the right iliac incision and the lateral thigh incision suggested that the primary infection site might be in the right iliac region. The implant bone acted as an indirect source of infection, leading to the secondary infection of the right femoral implant site.

There are 2 main treatment methods for implant-related infections. The first method includes debridement, antibiotic therapy, and implant retention. The second method involves debridement, followed by either implant removal or replacement, and antibiotic therapy.^[5]^ Given that the benefits of stability outweigh the risks of infection, implants should be retained during debridement in cases of early or acute infections. In cases of late infections, implant removal is recommended if feasible.^[5,8]^ Therefore, during the child’s first surgery at our hospital, we did not remove the internal fixation device of the femur, but removed it after the femur had healed well.

The patient experienced recurrent wound discharge for a period of 4 months. Despite undergoing multiple debridements, catheter drainage, and antibiotic therapy, the infection remained uncontrolled. We believe that this is related to the formation of a bacterial biofilm on the surface of the implants. The bacteria within this biofilm are resistant to antibiotics, increasing their resistance to conventional antibiotics by approximately 1000 times,^[9]^ which makes infections difficult to treat.^[10]^ When antibiotic concentrations decrease, these dormant bacteria can become activated, leading to recurrent and chronic infections. The incision infection in this case occurred more than 2 weeks after the patient’s discharge from the hospital. We speculated that this may be related to the antibiotic concentration. During hospitalization, intravenous antibiotics maintained a high drug concentration. After discharge, the patient switched to low-concentration oral antibiotics, which led to a recurrence of the infection. The patient was switched to oral antibiotics after 3 weeks of intravenous antibiotics in our hospital, but the treatment course was still insufficient. Therefore, the duration of antibiotic treatment needs to be extended. Literature recommends a duration of 4 to 6 weeks as appropriate.^[11]^

Existing antibiotics, such as vancomycin, linezolid, and teicoplanin, are effective against MRSA-related implant infections. However, these drugs suffer from poor targeting and antibiotic penetration. Many potential solutions have been proposed as remedies. Bone-bacteria dual-targeting nanoparticles composed of D6 and UBI peptides offer a promising approach for targeted antibiotic delivery to bone infections in infected rats, enhancing both antibiotic penetration and efficacy against bacterial pathogens.^[12]^ High-intensity focused ultrasound (HIFU)-induced thermal effects have been shown to enhance antibiotic concentrations in bone infection sites.^[13]^ Degradable coatings with antibacterial properties, such as Zn–Cu, Mg–Cu, Mg–Zn, and Zn–Ag alloys, inhibit the adhesion of MRSA and the subsequent formation of biofilms.^[14,15]^ However, the aforementioned therapeutic effects have been confirmed only through in vitro experiments or animal studies, and further research is needed to investigate the therapeutic effects of these new treatments in vivo.

As a single-center investigation, this study is inherently limited by its small sample size. Although dynamic radiographic monitoring successfully documented the implanted bone displacement process, the proposed abscess-driven migration mechanism remains hypothetical. This hypothesis requires validation through either biomechanical modeling experiments or large-scale multicenter clinical studies. Concerning antimicrobial therapy, although susceptibility-directed antibiotics (including vancomycin) were appropriately administered, 2 significant pharmacological limitations emerged: inadequate drug targeting specificity and suboptimal osseous tissue penetration. To address these challenges, future research should focus on 2 key directions: evaluating nanoparticle-based drug delivery systems for targeted therapy of infected osseous tissue, and investigating antimicrobial-coated implants for the prevention of device-related infections. The most substantial methodological limitation involves the lack of biofilm analysis on retrieved implants. We strongly recommend that subsequent studies integrate high-resolution scanning electron microscopy for biofilm morphological characterization with advanced microbiomic technologies for comprehensive microbial composition analysis. These approaches would provide definitive evidence of biofilm formation and enable data-driven optimization of anti-infective treatment protocols.

## 4. Conclution

The treatment of implant-related infections is complex and frequently necessitates a multidisciplinary approach. Hence, it is particularly important to prevent infections during the perioperative period. When an infection arises, clinicians must comprehensively assess the patient’s condition to ascertain the optimal timing for the removal of the implant. Additionally, meticulous imaging assessments should be carried out to determine whether there is any migration of infected bone along the abscess tract, thereby preventing reoperations necessitated by residual implants. Regarding the treatment of MRSA bacterial biofilms, while numerous novel drugs and therapeutic strategies documented in the literature have demonstrated effectiveness in vitro or in animal models，further clinical trials remain necessary to establish their feasibility and efficacy in vivo.

## Author contributions

**Conceptualization:** Xiao Qi Chen.

**Methodology:** Xiao Qi Chen.

**Investigation:** Xiao Qi Chen, Shi Hang Xu.

**Writing—original draft preparation:** Xiao Qi Chen.

**Writing—review and editing:** Xiao Qi Chen, Shu Ping Ma, Yue Han Li, Shi Hang Xu.

**Supervision:** Shi Hang Xu.
